# Age and Phenotype of Patients With Plaque Erosion

**DOI:** 10.1161/JAHA.120.020691

**Published:** 2021-09-25

**Authors:** Makoto Araki, Taishi Yonetsu, Osamu Kurihara, Akihiro Nakajima, Hang Lee, Tsunenari Soeda, Yoshiyasu Minami, Takumi Higuma, Shigeki Kimura, Masamichi Takano, Bryan P. Yan, Tom Adriaenssens, Niklas F. Boeder, Holger M. Nef, Chong Jin Kim, Iris McNulty, Filippo Crea, Tsunekazu Kakuta, Ik‐Kyung Jang

**Affiliations:** ^1^ Cardiology Division Massachusetts General Hospital Harvard Medical School Boston MA; ^2^ Biostatistics Center Massachusetts General Hospital Harvard Medical School Boston MA; ^3^ Department of Interventional Cardiology Tokyo Medical and Dental University Tokyo Japan; ^4^ Department of Cardiovascular Medicine Nara Medical University Kashihara Nara Japan; ^5^ Department of Cardiovascular Medicine Kitasato University School of Medicine Sagamihara Kanagawa Japan; ^6^ Division of Cardiology Department of Internal Medicine St. Marianna University School of Medicine Kanagawa Japan; ^7^ Division of Cardiology Kameda Medical Center Chiba Japan; ^8^ Cardiovascular Center Nippon Medical School Chiba Hokusoh Hospital Inzai Chiba Japan; ^9^ Department of Medicine and Therapeutics Faculty of Medicine The Chinese University of Hong Kong Hong Kong; ^10^ Department of Cardiovascular Medicine University Hospitals Leuven Leuven Belgium; ^11^ Department of Cardiology University of Giessen Giessen Germany; ^12^ Division of Cardiology Kyung Hee University Hospital Seoul South Korea; ^13^ Fondazione Policlinico Universitario A Gemelli IRCCS Roma Italy; ^14^ Department of Cardiology Tsuchiura Kyodo General Hospital Tsuchiura Ibaraki Japan

**Keywords:** acute coronary syndrome, age, cholesterol crystal, lipid‐rich plaque, plaque erosion, Acute Coronary Syndromes, Coronary Artery Disease, Optical Coherence Tomography (OCT)

## Abstract

**Background:**

A recent study reported that the outcome of patients with plaque erosion treated with stenting is poor when the underlying plaque is lipid rich. However, the detailed phenotype of patients with plaque erosion, particularly as related to different age groups, has not been systematically studied.

**Methods and Results:**

Patients with acute coronary syndromes caused by plaque erosion were selected from 2 data sets. Demographic, clinical, angiographic, and optical coherence tomography findings of the culprit lesion were compared between 5 age groups. Among 579 erosion patients, male sex and current smoking were less frequent, and hypertension, diabetes, and chronic kidney disease were more frequent in older patients. ST‐segment–elevation myocardial infarction was more frequent in younger patients. Percentage of diameter stenosis on angiogram was greater in older patients. The prevalence of lipid‐rich plaque (27.3% in age <45 years and 49.4% in age ≥75 years, *P*<0.001), cholesterol crystal (3.9% in age <45 years and 21.8% in age ≥75 years, *P*=0.027), and calcification (5.5% in age <45 years and 54.0% in age ≥75 years, *P*<0.001) increased with age. After adjusting risk factors, younger patients were associated with the presence of thrombus, and older patients were associated with greater percentage of diameter stenosis and the presence of lipid‐rich plaque and calcification.

**Conclusions:**

The demographic, clinical, angiographic, and plaque phenotypes of patients with plaque erosion distinctly vary depending on age. This may affect the clinical outcome in these patients.

**Registration:**

URL: https://www.clinicaltrials.gov. Unique identifiers: NCT03479723, NCT02041650.

**Clinical Perspective**

**What Is New?**

- This study investigated demographic, clinical, angiographic, and plaque phenotype of patients with erosion in different age groups.
- In patients with plaque erosion, advanced age is associated with higher prevalence of coronary risk factors, greater plaque burden, and more features of vulnerability.
- After adjusting coronary risk factors, stenosis severity, and the presence of lipid‐rich plaque and calcification are associated with advanced age.

**What Are the Clinical Implications?**

- Patients of advanced age with erosion may benefit from more intense cholesterol lowering and anti‐inflammatory therapy.

Although the pathophysiology of plaque rupture is well established, the mechanisms leading to plaque erosion remain less well understood.^1^ Medical therapy has proven effective for the stabilization of lipid‐rich atheromatous plaques, which are prone to rupture. However, targeted treatments for plaque erosion have not been established.^2^ Pathology studies have suggested that erosion occurs not only over lesions rich in smooth muscle cells and proteoglycans but also over lesions with lipid components.^3^, ^4^ A recent study showed that the outcome of percutaneous coronary intervention is poor in patients with erosion when the underlying plaque phenotype is lipid rich.^5^ Another study suggested that conservative therapy with antithrombotic therapy may be an option in selected patients with plaque erosion. Better understanding of plaque phenotype underneath erosion may help elucidate the mechanism of plaque erosion, predict the outcome, and establish targeted treatments.

Coronary artery disease associates strongly with age.^6^ The aim of this study was to investigate demographic, clinical, angiographic, and plaque phenotypes of patients with erosion in different age groups.

## Methods

The data that support the findings of this study are available from the corresponding author upon reasonable request.

### Study Population

Patients presenting with acute coronary syndrome (ACS) who underwent optical coherence tomography (OCT) imaging of the culprit lesion were selected from the Predictor (Identification of Predictors for Coronary Plaque Erosion in Patients with Acute Coronary Syndrome) study (NCT03479723) and the EROSION (Effective Anti‐Thrombotic Therapy Without Stenting: Intravascular Optical Coherence Tomography–Based Management In Plaque Erosion) study (NCT02041650). The Predictor study is an international, multicenter registry study that included patients with ACS undergoing OCT at 11 institutions in 6 countries (Japan, China, Italy, Belgium, United States, and Germany)^7^ from October 2008 to January 2018. The EROSION study is a single‐center, prospective, single‐arm study that included patients with ACS undergoing OCT from August 2014 to April 2016.^8^ Diagnosis of ACS, which included ST‐segment–elevation myocardial infarction (STEMI) and non‐ST‐segment–elevation acute coronary syndrome (NSTE‐ACS), was made according to the current American Heart Association/American College of Cardiology guidelines^9^, ^10^ as follows. STEMI was defined as continuous chest pain that lasted >30 minutes, arrival at the hospital within 12 hours from the onset of symptoms, ST‐segment elevation >0.1 mV in ≥2 contiguous leads or new left bundle‐branch block on the 12‐lead electrocardiogram, and elevated cardiac markers (creatine kinase‐MB or troponin I).^9^ NSTE‐ACS included non‐ST‐segment–elevation myocardial infarction and unstable angina. The former was defined as ischemic symptoms in the absence of ST‐segment elevation on the electrocardiogram with elevated cardiac markers. Unstable angina was defined as having newly developed/accelerating chest symptoms on exertion or rest angina within 2 weeks without biomarker release.^10^ Demographic and clinical data were collected at each participating site and sent to Massachusetts General Hospital (Boston, MA). Definitions of coronary risk factors are detailed in the Supplemental Methods (Data S1). The Predictor study and the EROSION study were approved by the institutional review boards at each participating site. For the Predictor study, informed consent was waived. For the EROSION study, written informed consent was obtained before enrollment.

Among the initial population of 1906 patients who had pre‐percutaneous coronary intervention culprit lesions imaged and had complete data, cases with stent‐related events (n=61), graft failure (n=3), incomplete data (n=54), and suboptimal image quality due to blood artifact, a short pullback, or massive thrombus (n=152) were excluded from this study. Among 1636 patients with ACS suitable for culprit lesion evaluation, plaque erosion was identified in 579 subjects (35.4%), who constituted the final study population (Figure S1).

### Angiographic Analysis

Coronary angiograms were analyzed with the Cardiovascular Angiography Analysis System (Pie Medical Imaging B.V., Maastricht, The Netherlands). The minimum lumen diameter, reference lumen diameter, lesion length, and percentage of diameter stenosis were measured. The distance from the respective coronary ostium to the culprit lesion was measured in the least foreshortened view on angiograms as previously described.^11^, ^12^ Initial Thrombolysis in Myocardial Infarction flow grade was also evaluated for the culprit vessel.

### OCT Image Acquisition

OCT examination was performed using either a frequency‐domain (81.3%) (C7/C8, OCT Intravascular Imaging System, St. Jude Medical, St. Paul, MN) or a time‐domain (18.7%) (M2/M3 Cardiology Imaging Systems, LightLab Imaging Inc., Westford, MA) OCT system. OCT imaging was performed before any percutaneous coronary intervention procedures, except aspiration thrombectomy for occlusive thrombus precluding visualization of underlying plaque. All OCT images were submitted to the core laboratory at Massachusetts General Hospital and analyzed by 2 independent investigators who were blinded to patients’ data, using an offline review workstation (St. Jude Medical). Any discordance was resolved by consensus with a third reviewer.

### OCT Image Analysis

**Figure 1:**
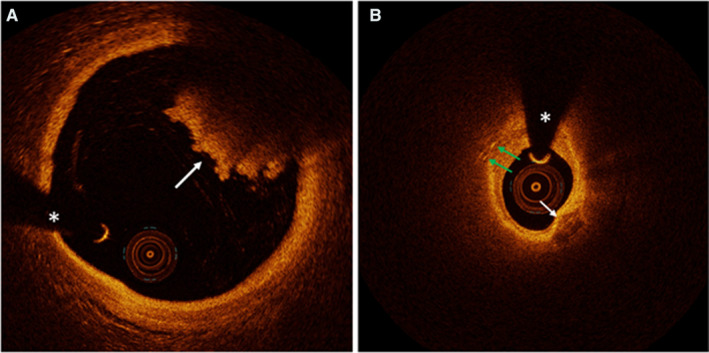
Representative OCT images in patients of different age. The asterisks indicate a guide wire artifact. **A**, The culprit lesion of a 37‐year‐old patient who presented with STEMI. The white arrow indicates a red thrombus. Lipid‐rich plaque, cholesterol crystal, and calcification were not observed. Because the red thrombus obscured the underlying area, the underlying plaque morphology could not be assessed in the area. **B**, The culprit lesion of a 77‐year‐old patient who presented with unstable angina. Lipid was observed from 6 o’clock to 11 o’clock. The green arrows indicate cholesterol crystals. The white arrow indicates calcification. OCT indicates optical coherence tomography; and STEMI ST‐segment–elevation myocardial infarction.

Plaque rupture was defined by the presence of fibrous cap discontinuity with a communication between the lumen and the inner core of plaque or with a cavity formed within the plaque.^8^, ^13^ Plaque erosion was identified by the presence of attached thrombus overlying an intact plaque, luminal surface irregularity at the culprit lesion in the absence of thrombus, or attenuation of the underlying plaque by thrombus without superficial lipid or calcification immediately proximal or distal to the site of thrombus.^8^, ^13^ Nearby bifurcation was predefined, when plaque erosion was identified within 5 mm proximal or distal to a side branch with an orifice diameter >1.0 mm measured by OCT.^12^, ^14^ Minimal lumen area site was chosen for the measurement of the distance between plaque erosion and the nearby bifurcation.^12^, ^14^ Representative OCT images are shown in Figure 1. Definitions of other OCT findings are detailed in the Supplemental Methods (Data S1).

### Statistical Analysis

Patients were categorized into 5 groups based on their age (years): <45, 45 to 54, 55 to 64, 65 to 74, and ≥75.^15^ Patient characteristics, angiographic findings, and plaque morphologies underneath erosion were compared between the age groups. Continuous variables with normal distribution were expressed as mean±SD, and median (interquartile range) was used to summarize nonnormally distributed variables. Categorical data were expressed as absolute frequencies and percentages. Global trends by age were assessed using the Jonckheere‐Terpstra trend test for continuous variables and using the Cochran‐Armitage trend test for categorical data. Furthermore, data were analyzed after adjusting confounding characteristics of sex, current smoking, hypertension, dyslipidemia, diabetes, chronic kidney disease, previous myocardial infarction, previous percutaneous coronary intervention, estimated glomerular filtration rate, total cholesterol, low‐density lipoprotein cholesterol, high‐density lipoprotein cholesterol, triglycerides, and hemoglobin A1c using multivariate logistic regression. Age was considered as a continuous variable rather than a categorical variable in this analysis. Multivariable linear regression was used to adjust confounding factors in continuous angiography and OCT variables. Sensitivity analyses were performed to investigate whether the results were maintained in patients with STEMI and patients with acute myocardial infarction including STEMI or non‐ST‐segment–elevation MI. Because the definition of plaque erosion by OCT was not identical to the definition from pathology study,^3^ the data were also analyzed after excluding patients in whom thrombus was not observed or smaller than the diagnostic criteria (250 µm).

Intra‐ and interobserver reliability for OCT diagnoses were assessed by kappa statistics. A 2‐sided *P* value of <0.05 was considered statistically significant. Statistical analysis was performed using R software version 3.6.2 (R Foundation for Statistical Computing, Vienna, Austria).

## Results

### Clinical Characteristics

Among 579 patients with plaque erosion, 298 (51.5%) presented with STEMI and 281 (48.5%) with non‐ST‐segment acute coronary syndrome (NSTE‐ACS). Mean age was 61.3 years with male predominance (80.0%). Baseline characteristics of patients in each age group are summarized in Table 1. The proportion of men (92.7% in age <45 years to 70.1% in age ≥75 years) and current smokers (63.6% in age <45 years to 16.1% in age ≥75 years), and body mass index (25.9±3.7 kg/m^2^ in age <45 years to 23.5±3.0 kg/m^2^ in age ≥75 years) significantly decreased with advanced age. The prevalence of hypertension (38.2% in age <45 years to 73.6% in age ≥75 years), diabetes (16.4% in age <45 years to 35.6% in age ≥75 years), and chronic kidney disease (7.3% in age <45 years to 21.8% in age ≥75 years) significantly increased with age. STEMI was the predominant type of presentation in younger patients, whereas NSTE‐ACS became more frequent in older patients (STEMI: 61.8% in age <45 years and 34.5% in age ≥75 years). Older patients were more frequently taking antihypertensive medications on admission (angiotensin‐converting enzyme inhibitor or angiotensin II receptor blocker: 12.5% in age <45 years and 39.3% in age ≥75 years; calcium channel inhibitor: 15.4% in age <45 years and 39.3% in age ≥75 years). Estimated glomerular filtration rate (76.1±24.4 mL/min per 1.73 m^2^ in age <45 years and 66.6±24.1 mL/min per 1.73 m^2^ in age ≥75 years) and hemoglobin levels (15.1±1.4 g/dL in age <45 years and 13.5±1.9 g/dL in age ≥75 years) were lower, and high‐density lipoprotein cholesterol levels (45.5±13.6 mg/dL in age <45 years to 48.1±11.0 mg/dL in age ≥75 years) were higher in advanced age groups.

**Table 1:** Patient Characteristics

	Age, y					
	<45 (n=55)	45–54 (n=119)	55–64 (n=158)	65–74 (n=160)	≥75 (n=87)	*P* value
Male sex	51 (92.7)	104 (87.4)	130 (82.3)	117 (73.1)	61 (70.1)	<0.001
Body mass index, kg/m^2^	25.9±3.7	25.4±3.8	25.2±2.8	24.4±3.8	23.5±3.0	<0.001
Current smoking	35 (63.6)	75 (63.0)	88 (55.7)	60 (37.5)	14 (16.1)	<0.001
Hypertension	21 (38.2)	50 (42.0)	76 (48.1)	101 (63.1)	64 (73.6)	<0.001
Dyslipidemia	22 (40.0)	74 (62.2)	89 (56.3)	94 (58.8)	54 (62.1)	0.097
Diabetes	9 (16.4)	28 (23.5)	35 (22.2)	42 (26.3)	31 (35.6)	0.011
Chronic kidney disease	4 (7.3)	10 (8.4)	13 (8.2)	22 (13.8)	19 (21.8)	0.001
Previous myocardial infarction	3 (5.5)	6 (5.0)	12 (7.6)	5 (3.1)	5 (5.7)	0.686
Previous percutaneous coronary intervention	2 (3.6)	5 (4.2)	16 (10.1)	11 (6.9)	7 (8.0)	0.246
Previous coronary artery bypass graft	0 (0.0)	0 (0.0)	1 (0.6)	1 (0.6)	0 (0.0)	0.706
Clinical presentation						<0.001
ST‐segment–elevation myocardial infarction	34 (61.8)	66 (55.5)	91 (57.6)	77 (48.1)	30 (34.5)	
Non‐ST‐segment–elevation acute coronary syndrome	21 (38.2)	53 (44.5)	67 (42.4)	83 (51.9)	57 (65.5)	
Medication						
Aspirin	6 (25.0)	12 (14.3)	26 (26.5)	20 (19.6)	10 (16.4)	0.785
P2Y12 inhibitor	3 (12.5)	9 (10.7)	11 (11.2)	11 (10.7)	6 (9.8)	0.768
Statin	3 (12.5)	15 (17.9)	27 (27.6)	26 (25.2)	10 (16.7)	0.587
Beta blocker	7 (29.2)	10 (11.9)	15 (15.5)	22 (21.4)	5 (8.2)	0.404
Angiotensin‐converting enzyme inhibitor or angiotensin II receptor blocker	3 (12.5)	18 (21.4)	31 (31.6)	28 (27.2)	24 (39.3)	0.010
Calcium channel inhibitor	2 (15.4)	7 (11.5)	22 (26.5)	26 (28.6)	22 (39.3)	<0.001
Laboratory data						
Estimated glomerular filtration rate, mL/min per 1.73 m^2^	76.1±24.4	72.7±26.1	69.9±20.5	68.0±19.8	66.6±24.1	0.005
Total cholesterol, mg/dL	181.1±46.6	193.0±47.9	181.2±43.2	191.2±39.7	184.1±38.4	0.693
Low‐density lipoprotein cholesterol, mg/dL	117.3±43.6	123.8±43.7	116.5±42.2	123.5±37.0	115.2±36.0	0.817
High‐density lipoprotein cholesterol, mg/dL	45.5±13.6	46.9±12.5	46.1±14.3	48.7±13.8	48.1±11.0	0.023
Triglycerides, mg/dL	117.0 (47.0–196.9)	104.8 (61.2–159.6)	115.6 (67.0–160.1)	104.0 (69.2–160.5)	87.0 (60.5–143.0)	0.178
Hemoglobin A1c, %	6.4±2.0	6.3±1.4	6.2±1.4	6.1±1.0	6.2±1.1	0.389
High‐sensitivity C‐reactive protein, mg/dL	0.33 (0.10–0.82)	0.20 (0.08–0.45)	0.24 (0.07–0.69)	0.10 (0.03–0.45)	0.30 (0.06–0.72)	0.345
Hemoglobin, g/dL	15.1±1.4	14.6±1.6	14.4±1.6	14.1±1.7	13.5±1.9	<0.001
Peak creatine kinase‐MB, IU/L	80.8 (17.2–251.1)	93.0 (22.3–227.0)	97.8 (16.3–237.4)	64.1 (17.3–263.3)	70.0 (18.0–170.0)	0.358
Left ventricular ejection fraction, %	58.1±7.3	57.4±11.0	56.7±10.3	58.0±10.8	56.0±12.1	0.787

*P* values are for the Jonckheere‐Terpstra trend test for continuous variables or the Cochran‐Armitage trend test for categorical data. Medication data were analyzed only in available cases.

### Angiographic Findings

The angiographic features of culprit lesions are summarized in Table 2. The distribution of culprit vessels (right coronary artery, left anterior descending artery, or left circumflex) was comparable among the age groups. Plaque erosions were clustered in the proximal coronary artery, particularly in the left anterior descending artery. The mean distance from the coronary ostium to plaque erosion was similar among the age groups (31.4, 32.6, 29.8, 32.4, and 33.7 mm in the age groups of <45, 45–54, 55–64, 65–74 and ≥75, respectively). Initial Thrombolysis in Myocardial Infarction flow grade ≤1 was more frequent in younger patients (36.4% in age <45 years and 19.8% in age ≥75 years). Minimum lumen diameter (1.35±0.82 mm in age <45 years and 0.59±0.52 mm in age ≥75 years) and reference lumen diameter (3.48±0.73 mm in age <45 years and 2.70±0.68 mm in age ≥75 years) were significantly smaller, and percentage of diameter stenosis (61.5±20.2% in age <45 years and 77.9±18.9% in age ≥75 years) was significantly greater in older patients. Percentage of diameter stenosis was more than 70% in 32.7% of patients aged <45 years and in 62.1% of patients aged ≥75 years.

**Table 2:** Angiographic Findings

	Age, y					
	<45 (n=55)	45–54 (n=119)	55–64 (n=158)	65–74 (n=160)	≥75 (n=87)	*P* value
Infarct‐related artery						0.450*
RCA	13 (23.6)	30 (25.2)	42 (26.6)	57 (35.6)	24 (27.6)	
LAD	37 (67.3)	71 (59.7)	95 (60.1)	80 (50.0)	51 (58.6)	
LCx	5 (9.1)	18 (15.1)	21 (13.3)	23 (14.4)	12 (13.8)	
Culprit lesion site						0.891*
Proximal segment	22 (40.0)	45 (39.1)	69 (44.2)	68 (43.3)	38 (44.2)	
Mid segment	20 (36.4)	46 (40.0)	51 (32.7)	60 (38.2)	27 (31.4)	
Distal segment	13 (23.6)	24 (20.9)	36 (23.1)	29 (18.5)	21 (24.4)	
Multivessel disease	16 (29.1)	32 (27.8)	52 (34.4)	55 (35.5)	30 (35.7)	0.164
Initial Thrombolysis in Myocardial Infarction flow ≤1	20 (36.4)	46 (40.0)	59 (37.8)	51 (32.5)	17 (19.8)	0.007
Distance from the ostium, mm	31.4±21.8	32.6±18.1	29.8±19.2	32.4±21.0	33.7±24.5	0.920
RCA	48.8±24.2	39.4±19.8	39.4±24.6	43.8±26.3	56.8±30.2	0.206
LAD	24.7±18.8	29.3±16.6	24.4±14.5	24.9±13.3	22.7±12.8	0.372
LCx	34.0±4.6	34.9±18.9	34.7±17.0	29.7±14.8	30.4±14.1	0.255
Quantitative coronary angiography data						
Minimum lumen diameter, mm	1.35±0.82	0.73±0.61	0.79±0.63	0.68±0.64	0.59±0.52	<0.001
Reference vessel diameter, mm	3.48±0.73	2.85±0.66	2.99±0.70	2.84±0.55	2.70±0.68	<0.001
Lesion length, mm	14.2±5.5	15.1±6.9	14.6±6.2	14.6±6.3	15.6±6.9	0.442
Diameter stenosis, %	61.5±20.2	75.1±20.4	74.0±19.4	76.9±18.9	77.9±18.9	<0.001
Diameter stenosis >70%	18 (32.7)	68 (57.1)	81 (51.3)	102 (66.2)	54 (62.1)	<0.001

*P* values are for the Jonckheere‐Terpstra trend test for continuous variables or the Cochran‐Armitage trend test for categorical data. Angiographic data except infarct‐related artery were missing in 10 (1.7%) cases. LAD indicates left anterior descending artery; LCx, left circumflex artery; and RCA, right coronary artery.

* *P* value for χ^2^ test.

### OCT Findings

**Figure 2:**
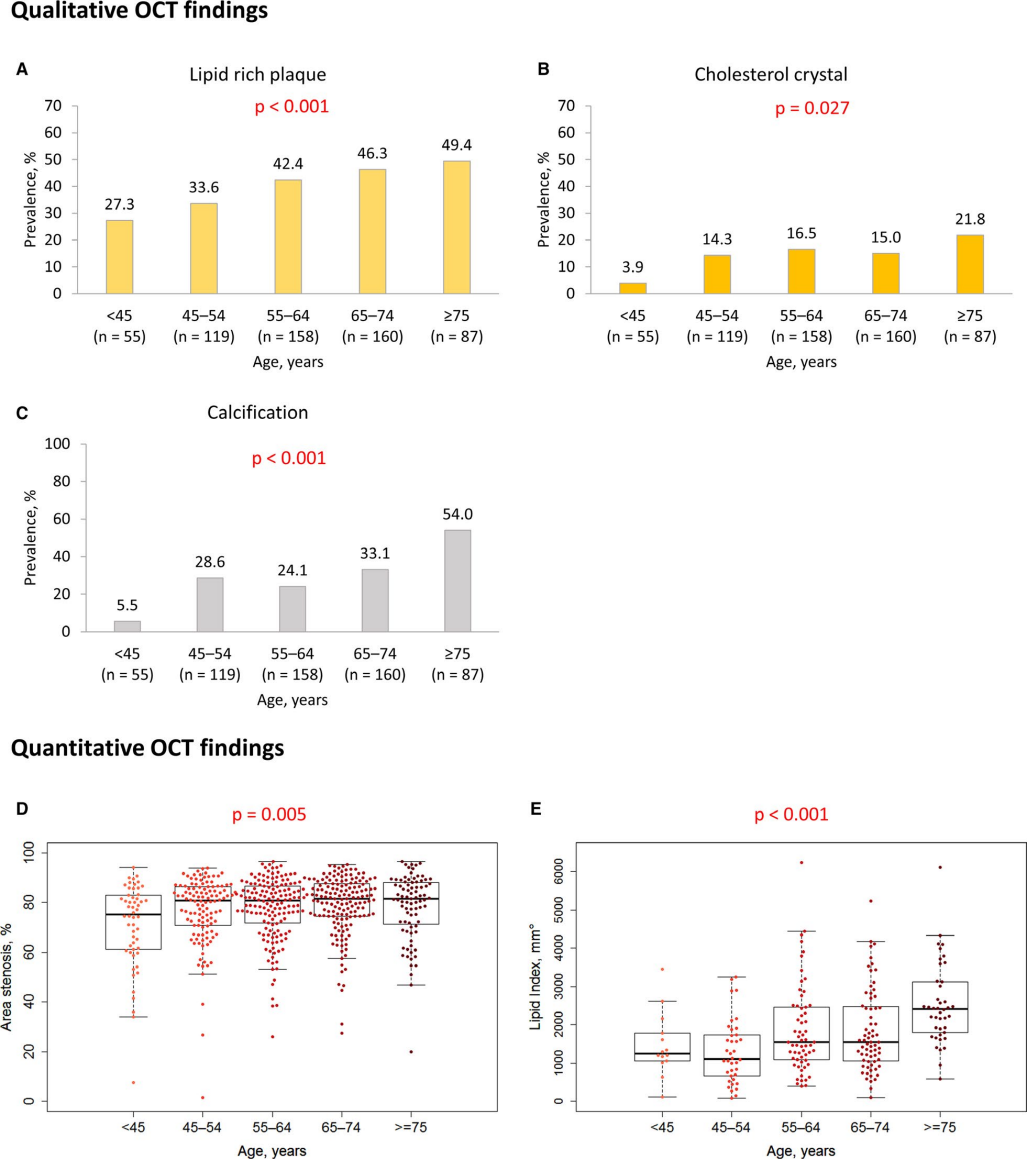
Comparison of plaque phenotype between different age groups. The prevalence of (**A**) lipid‐rich plaque, (**B**) cholesterol crystal, and (**C**) calcification were significantly more frequently observed in older age groups. Beeswarm plots, and box and whisker plots show quantitative OCT findings in different age groups (**D** and **E**). (**D**) Percentage of area stenosis and (**E**) lipid index were significantly greater in older age groups. *P* values are for trend tests. OCT indicates optical coherence tomography.

The prevalence of lipid‐rich plaque increased with age from 27.3% in age <45 years to 49.4% in age ≥75 years (*P*<0.001). The prevalence of cholesterol crystal and calcification also significantly increased with age (from 3.9% to 21.8% and from 5.5% to 54.0%, respectively). The prevalence of thrombus decreased with increasing age, appearing in 89.1% in age <45 years to 62.1% in age ≥75 years (*P*<0.001) (Table 3), consistent with more frequent NSTE‐ACS in older age groups. There was no statistically significant age‐related difference in the prevalence of proximity to bifurcation. Minimum lumen area (1.92 [1.20–3.74] mm^2^ in age <45 years and 0.99 [0.73–1.30] mm^2^ in age ≥75 years) and reference lumen area (8.67 [6.49–10.46] mm^2^ in age <45 years and 5.74 [4.23–7.16] mm^2^ in age ≥75 years) were significantly smaller (Table 3), and percentage of area stenosis (75.2 [61.3–82.7]% in age <45 years and 81.4 [71.6–88.0]% in age ≥75 years) was significantly greater in older patients. Mean lipid arc (median: 184.7° in age <45 years and 251.5° in age ≥75 years), lipid length (median: 7.3 mm in age <45 years and 9.7 mm in age ≥75 years) (Table 3), and lipid index (median: 1248.9°mm in age <45 years and 2410.0°mm in age ≥75 years) were significantly greater in older patients. Minimum fibrous cap thickness was comparable among the groups (*P*=0.36). The intraobserver kappa coefficients for diagnoses of lipid‐rich plaque, cholesterol crystal, and calcification were 0.91, 0.87, and 0.82, respectively. The interobserver kappa coefficients for diagnoses of lipid‐rich plaque, cholesterol crystal, and calcification were 0.82, 0.87, and 0.91, respectively (Figure 2 and Table 3).

**Table 3:** OCT Findings in Different Age Groups

	Age, y					
	<45 (n=55)	45–54 (n=119)	55–64 (n=158)	65–74 (n=160)	≥75 (n=87)	*P* value
Qualitative						
Lipid‐rich plaque	15 (27.3)	40 (33.6)	67 (42.4)	74 (46.3)	43 (49.4)	<0.001
Thin‐cap fibroatheroma	1 (1.8)	8 (6.7)	11 (7.0)	11 (6.9)	9 (10.3)	0.144
Cholesterol crystal	2 (3.9)	17 (14.3)	26 (16.5)	24 (15.0)	19 (21.8)	0.036
Calcification	3 (5.5)	34 (28.6)	38 (24.1)	53 (33.1)	47 (54.0)	<0.001
Thrombus	49 (89.1)	105 (88.2)	119 (75.3)	114 (71.3)	54 (62.1)	<0.001
White	38 (77.6)	76 (72.4)	100 (84.0)	94 (82.5)	38 (70.4)	0.764
Red	11 (22.4)	29 (27.6)	19 (16.0)	20 (17.5)	16 (29.6)	
Nearby bifurcation	19 (34.5)	39 (32.8)	56 (35.4)	43 (26.9)	22 (25.3)	0.091
Right coronary artery	0/13 (0.0)	4/30 (13.3)	6/42 (14.3)	8/57 (14.0)	4/24 (16.7)	0.274
Left anterior descending artery	18/37 (48.6)	30/71 (42.3)	42/95 (44.2)	29/80 (36.2)	16/51 (31.4)	0.069
Left circumflex artery	1/5 (20.0)	5/18 (27.8)	8/21 (38.1)	6/23 (26.1)	2/12 (16.7)	0.617
Quantitative						
Minimum lumen area, mm^2^	1.92 (1.20–3.74)	1.18 (0.80–1.84)	1.15 (0.80–1.72)	0.92 (0.77–1.55)	0.99 (0.73–1.30)	<0.001
Reference lumen area, mm^2^	8.67 (6.49–10.46)	6.29 (4.65–8.02)	6.50 (5.10–8.03)	5.88 (4.50–7.68)	5.74 (4.23–7.16)	<0.001
Area stenosis, %	75.2 (61.3–82.7)	80.8 (70.8–86.3)	80.7 (72.0–86.5)	81.6 (74.5–87.5)	81.4 (71.6–88.0)	0.005
Minimum fibrous cap thickness, µm	130.0 (80.0–156.5)	97.0 (70.0–130.0)	100.0 (78.5–135.0)	103.0 (80.0–134.0)	107.0 (80.0–141.5)	0.360
Mean lipid arc	184.7 (157.9–236.3)	191.7 (145.3–244.2)	212.6 (182.8–257.8)	200.1 (156.3–261.5)	251.5 (208.4–276.8)	<0.001
Lipid length, mm	7.3 (5.2–9.2)	6.6 (3.5–8.9)	7.9 (5.4–9.9)	8.4 (5.8–10.4)	9.7 (7.9–12.2)	<0.001
Lipid index, mm	1248.9 (1079.1–1745.2)	1109.8 (677.9– 1730.9)	1538.8 (1083.3–2460.1)	1553.1 (1063.1–2458.0)	2410.0 (1825.2–3097.3)	<0.001

*P* values are for the Jonckheere‐Terpstra trend test for continuous variables or the Cochran‐Armitage trend test for categorical data. OCT indicates optical coherence tomography.

### Risk‐Adjusted Analyses

**Figure 3:**
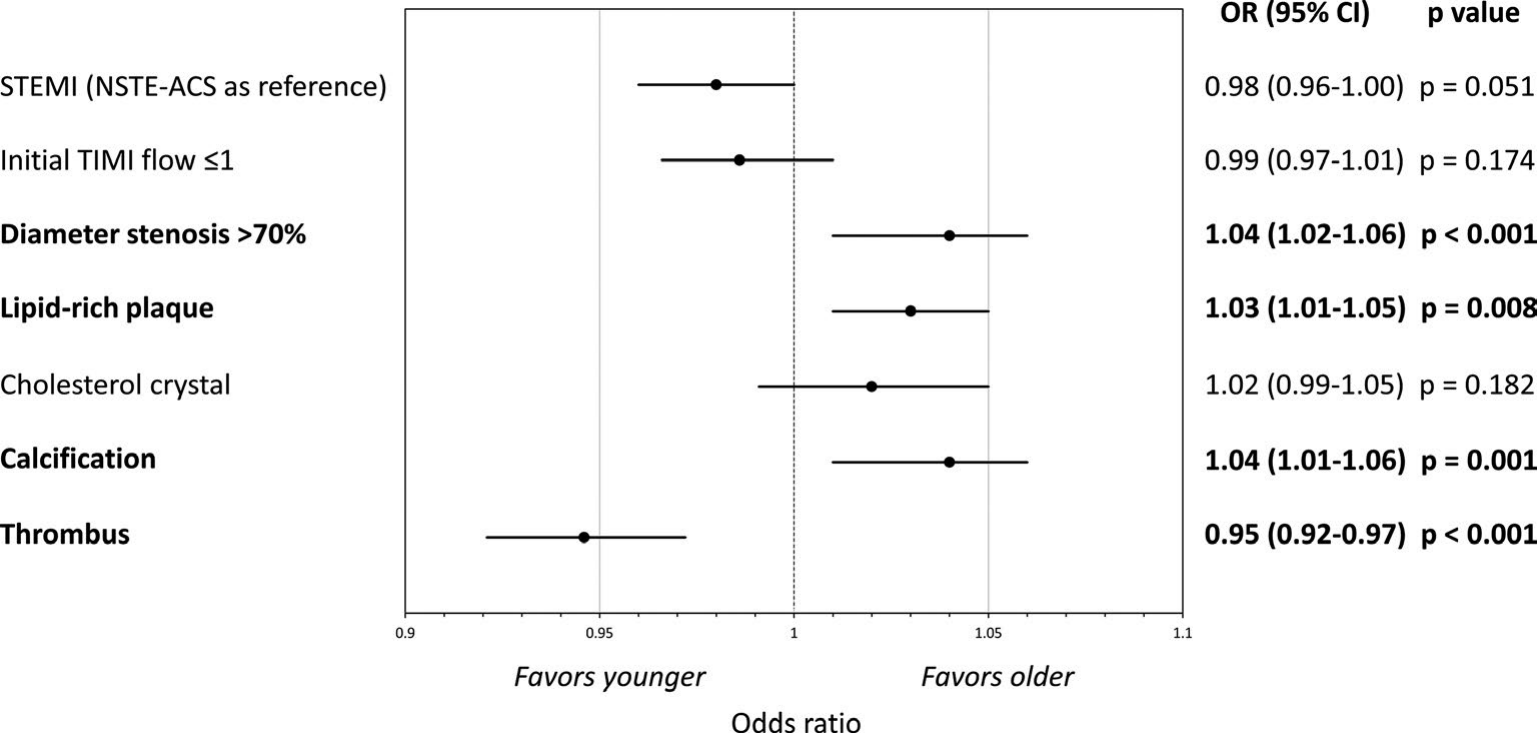
The impact of age on clinical presentation, stenosis severity, and lesion phenotype, adjusted for patient characteristics. After adjusting patient characteristics (sex, current smoking, hypertension, dyslipidemia, diabetes, chronic kidney disease, previous myocardial infarction, previous percutaneous coronary intervention, estimated glomerular filtration rate, total cholesterol, low‐density lipoprotein‐cholesterol, high‐density lipoprotein‐cholesterol, triglycerides, and hemoglobin A1c), younger patients were associated with the presence of thrombus, and older patients were associated with greater diameter stenosis and the presence of lipid‐rich plaque and calcification. The detail of each multivariate logistic regression is shown in Table S1. NSTE‐ACS indicates non‐ST‐segment elevation acute coronary syndrome; OR, odds ratio; STEMI, ST‐segment elevation myocardial infarction; and TIMI, Thrombolysis in Myocardial Infarction.

After adjusting risk factors, older patients were still associated with the absence of thrombus (odds ratio [OR], 0.95; 95% CI, 0.92–0.97; *P*<0.001), greater diameter stenosis (OR, 1.04; 95% CI, 1.02–1.06; *P*<0.001) and the presence of lipid‐rich plaque (OR, 1.03; 95% CI, 1.01–1.05; *P*=0.008) and calcification (OR, 1.04; 95% CI, 1.01–1.06; *P*=0.001) (Figure 3). Impacts of age on angiographic initial Thrombolysis in Myocardial Infarction flow ≤1 and the presence of cholesterol crystal were not statistically significant in the risk‐adjusted analyses. The detail of each multivariate logistic regression is shown in Table S1. After adjusting risk factors, minimum lumen diameter, reference lumen diameter, diameter stenosis, minimum lumen area, mean lipid arc, lipid length, and lipid index were still associated with age (Table S2).

### Sensitivity Analysis

Consistent age‐related differences in the distribution of sex, coronary risk factors and angiographic stenosis severity, the prevalence of lipid‐rich plaque, cholesterol crystal, calcification, and lipid burden were observed in patients with STEMI (Table S3) and in patients with acute myocardial infarction (combined STEMI and non‐ST‐segment–elevation myocardial infarction) (Table S4). After excluding 138 (23.8%) patients in whom thrombus was not observed or smaller than the diagnostic criteria (250 µm), most results remained unchanged (Table S5). In this subset, the prevalence of thin‐cap fibroatheroma significantly increased with age, which was not significant in the main analysis. Because the prevalence of thrombus may have been affected by aspiration thrombectomy before OCT imaging, the prevalence of thrombus was also analyzed after excluding those who underwent aspiration thrombectomy (Table S6). The results remained unchanged.

## Discussion

The collection of a large number of plaque erosion cases provided an opportunity to study detailed phenotypes of plaque erosion in subgroups of patients in different age categories. The main results of the current study show that with increasing age (1) the proportion of men decreases and coronary risk factors increase; (2) the relative incidence of NSTE‐ACS, compared with STEMI, increases; (3) stenosis severity on angiogram increases; and (4) features of plaque vulnerability including lipid‐rich plaque, cholesterol crystal, and calcification increase but fibrous cap thickness did not increase. After adjusting for coronary risk factors, stenosis severity and the presence of lipid‐rich plaque and calcification were associated with age.

### Age and Demographic Phenotype of Erosion

The development of coronary artery disease is strongly associated with age.^6^ Mehta et al^16^ investigated a large number of patients with acute myocardial infarction and reported a higher prevalence of hypertension, diabetes, and renal insufficiency and a lower proportion of men and current smokers in older patients. The results of the current study with a specific subgroup of patients with plaque erosion are consistent with Mehta’s study in that the proportion of men decreased and the prevalence of coronary risk factors increased with age. Although pathology studies with a small sample size have shown that plaque erosion was frequent in young women with sudden cardiac death, recent in vivo studies consistently have shown that the majority of patients with plaque erosion were male.^12^, ^13^ Notably, the current result showed that the proportion of women increased with age. Estrogen is known to have protective effects against atherosclerosis and thrombosis,^17^ mediated by endothelial estrogen receptor‐α.^18^ Estrogen withdrawal at menopause results in alterations in endothelial dysfunction, vascular inflammation, sympathetic tone, and a higher insulin resistance.^19^

### Age and Clinical and Angiographic Phenotype of Erosion

A previous STEMI study reported that age <50 years was associated with higher prevalence of plaque erosion. The present study included patients with ACS and plaque erosion only and divided them into 5 age groups to assess the impact of age on the phenotype of patients with plaque erosion including the age‐related difference in plaque morphology. The higher relative incidence of NSTE‐ACS in older patients has been reported in previous studies.^15^, ^16^, ^20^ In the present study, this finding was confirmed in patients with erosion as well. This result may be explained by evidence that repetitive ischemic insult by greater plaque burden induces ischemic preconditioning.^21^ In the present study, the prevalence of diameter stenosis >70% doubled in the oldest group, compared with the youngest group. The concept of individualizing the management of patients with ACS depending on the underlying pathology was tested in the EROSION study.^8^ The study showed that patients with plaque erosion were successfully managed with antithrombotic therapy without stenting. However, it should be acknowledged that patients with residual diameter stenosis >70% on angiogram after coronary thrombectomy were excluded from the study. Considering the results of the present study, younger patients with erosion may be a better target for conservative management.

### Age and Plaque Phenotype of Erosion

A previous pathology study suggested that coronary plaque burden gradually increases with age.^22^ In an intravascular ultrasound study, plaque burden, necrotic core, and calcium content were shown to increase with age.^20^ Recently, an OCT study assessed culprit lesion morphology in young patients with ACS and showed that patients aged ≤50 years less frequently had vulnerable plaque features.^23^ In the present study, we investigated only patients with erosion and found that vulnerable plaque phenotype increases with age in this subset. These differences may be explained by the increased prevalence of coronary risk factors in older patients. Recent research showed that age‐dependent endothelial dysfunction favors atherogenesis and thrombosis and predisposes to coronary events.^24^ Aged endothelial cells downregulate *JunD* and *SIRT1* expression, leading to pro‐oxidant and proinflammatory gene expression. As a consequence, increased reactive oxygen species and inflammatory cytokines reduce nitric oxide availability. In parallel, age‐related up‐regulation of angiotensin II and cyclooxygenase‐derived eicosanoids results in augmented endothelin‐1, thromboxane A_2_, and prostaglandin F_2_α. These mechanisms together could impair endothelial function and promote thrombosis in elderly people.

Although typical plaque erosion occurs over lesions rich in proteoglycans and smooth muscle cells with a local absence of intimal endothelial cells,^25^ it is also known that plaque erosion can occur over lesions with lipid components.^3^, ^4^ A recent in vivo case series also reported these 2 distinct phenotypes with multimodality imaging.^26^ In an OCT study that assessed the clinical significance of lipid‐rich plaque underneath erosion,^5^ the incidence of major adverse cardiac events, including cardiac death, myocardial infarction, and clinically driven revascularizations, was higher in patients with erosion and underlying lipid‐rich plaque who underwent percutaneous coronary intervention.

Several intravascular studies showed that statin therapy can stabilize lipid‐rich plaques.^27^, ^28^ The most recent guideline recommends intensive lipid management for secondary prevention of ACS.^29^ Yet, the significance of intensive lipid management for plaque erosion, especially in older patients, is unknown. Our results showed greater plaque vulnerability in older patients and suggest that intensive lipid management may be beneficial in this group.

### Mechanisms of Plaque Erosion in Elderly Patients

It has been discussed that the mechanism of plaque erosion is considerably different from that of plaque rupture.^2^ Disruption of fibrous cap overlying necrotic core triggers thrombosis in plaque rupture, whereas endothelial cell denudation triggers thrombosis in plaque erosion. Young women without coronary risk factors but with smoking habits were assumed to have a higher risk of plaque erosion. Plaque erosion showed less severe stenosis and lower lipid burden compared with plaque rupture. In the present study, the phenotype of older patients with plaque erosion was different from the historically assumed phenotype of patients with plaque erosion. They were nonsmokers and more frequently had hypertension, diabetes, chronic kidney disease, severe stenosis, lipid‐rich plaque, cholesterol crystal, calcification, thrombus, and large lipid burden than younger patients.

We hypothesized that older patients may have a distinct phenotype of plaque erosion. Pathology studies have suggested that 2 distinct phenotypes of plaque erosion may exist: lesions rich in smooth muscle cells and proteoglycans and lesions rich in lipid components.^3^, ^4^ This was also confirmed in a recent case series that assessed plaque phenotype underlying plaque erosion using OCT, near‐infrared spectroscopy–intravascular ultrasound, and coronary angioscopy and reported the presence of 2 distinct phenotypes of plaque erosion different in the extent of near‐infrared spectroscopy‐derived lipid core burden and coronary angioscopy‐derived luminal surface color.^26^

Stenosis severity of the culprit lesion was greater in older patients. It is known that tight stenosis causes higher shear stress.^30^ Therefore, it is possible that tight stenosis with high local shear stress triggered the process of plaque erosion^31^ in older patients. In the present study, cholesterol crystal was more frequently observed in older erosion patients. In addition, the proportion of women increased with age. Abela et al^32^ reported that cholesterol crystals may perforate the endothelial layer and cause plaque erosion if the lipid pool is relatively small. A pathology study reported that the prevalence of plaque erosion was higher in women than men.^33^ It was also reported that the volume of lipid pool in the carotid artery was smaller in women than men.^34^ It is possible that cholesterol crystal was involved in the mechanism of plaque erosion particularly in elderly women with relatively small lipid pools.

Several previous studies reported that plaque erosion is associated with smoking.^3^, ^7^, ^12^ Smoking promotes activation of both platelets and clotting factors.^35^ In addition, smoking causes endothelial damage^36^ as well as activation of rho‐kinase,^37^ which leads to vasoconstriction or vasospasm. These mechanisms may be particularly important in younger patients. Although it requires further corroboration in the future, plaque erosion may not be a unique entity but may have 2 distinct phenotypes depending on age.

### Limitations

Several limitations should be acknowledged in this study. First, although patients were prospectively enrolled in the registry at each institution, the present analysis was done retrospectively. Therefore, selection bias cannot be excluded. However, most patients were enrolled in institutions where OCT is routinely used. Therefore, unless there was a contraindication, the majority of consecutive patients were included in the study. Second, this study used 2 different OCT systems (time‐domain and frequency‐domain OCT), though time‐domain OCT use was in the minority of cases (18.7%). Both systems used light sources with the same center wavelength (1300 nm) and bandwidth, resulting in similar axial resolution (15 µm). Third, the hallmark of plaque erosion in pathology is the absence of endothelial monolayer. The axial resolution of OCT is not sufficient to detect the absence of endothelial cells. In addition, unlike pathology studies, patients have been treated with antithrombotic therapies before OCT imaging. This is why the specific algorithm for the diagnosis of plaque erosion by OCT was developed.^13^ This algorithm has been widely used in OCT studies since its first publication. Fourth, because of the shallow penetration depth of OCT, plaque burden or vessel remodeling could not be assessed. Fifth, it is possible that aspiration thrombectomy for occlusive thrombus affected lesion morphologies. Extreme care was exercised not to damage underlying plaque. Sixth, 23.8% of patients were diagnosed with plaque erosion based on an irregularity of the luminal surface. There is also a possibility that type 2 myocardial infarction caused by coronary spasm or embolism might have been included in the present study.^38^ However, the conclusions remained unchanged after excluding such patients without apparent thrombus. Finally, overlying massive thrombus can indeed hinder the accurate analysis of the underlying plaque structure. The predominant type of thrombus in plaque erosion is platelet‐rich. Light penetrates platelet‐rich thrombus and can visualize the underlying structure. That is why a diagnosis of plaque erosion can be made by OCT in the presence of residual thrombus in the majority of cases. In the present study, only 95 (16.4%) cases had red thrombus. Nonetheless, 152 patients were excluded because of suboptimal OCT image quality.

## Conclusions

The demographic, clinical, angiographic, and plaque phenotype of patients with plaque erosion is distinctly different depending on age. These phenotypes, which may be attributed to the difference in the pathophysiology, may affect the clinical outcome in erosion patients.

## Sources of Funding

Ik‐Kyung Jang’s research was supported by the Allan and Gill Gray Founation in Cardiology and by Mr and Mrs Michael and Kathryn Park.

## Disclosures

Ik‐Kyung Jang has received educational grants from Abbott Vascular and a consulting fee from Svelte. They had no role in the design or conduct of this research. The remaining authors have no disclosures to report.

## Supporting information

Data S1
Tables S1–S6
Figure S1
References 3, 39, 40, 41, 42, 43, 44, 45
