# Supplementary material for: Age and Phenotype of Patients With Plaque Erosion
Source: J Am Heart Assoc. 2021 Sep 25;10(19):e020691. doi: 10.1161/JAHA.120.020691 (PMC8649143; doi:10.1161/JAHA.120.020691)
Supplement: Supplementary file 1 — Data S1 Tables S1–S6 Figure S1 References 3, 39, 40, 41, 42, 43, 44, 45 [file JAH3-10-e020691-s001.pdf]

SUPPLEMENTARY MATERIALS

Data S1. Supplemental Methods

Definitions of Coronary Risk Factors

Hypertension was defined as systolic blood pressure ≥140 mmHg or diastolic blood pressure ≥90 mmHg or current use of anti-hypertensive treatment. Diabetes mellitus was diagnosed if a patient met 1 of the following criteria: documented history of diabetes mellitus, use of hypoglycemic agents, fasting glucose ≥126 mg/dL, 2-h plasma glucose level ≥200mg/dL in the oral glucose tolerance test, classic symptom with casual plasma glucose level ≥200 mg/dL, or hemoglobin A1c (HbA1c) ≥6.5%. Dyslipidemia was defined as total cholesterol (TC) level ≥220 mg/dL, triglycerides ≥150 mg/dL, low-density lipoprotein cholesterol (LDL-C) ≥140 mg/dL, high-density lipoprotein cholesterol (HDL-C) ≤40 mg/dL or taking medication for dyslipidemia. The glomerular filtration rate (eGFR) was calculated by using Chronic Kidney Disease Epidemiology Collaboration (CKD-EPI) equation,[39] and chronic kidney disease was defined as eGFR <60 $mL/min/1.73m^2$.

OCT Image Analysis

Lipid arc was measured at 1-mm intervals and lipid-rich plaque was defined as a plaque that had maximum lipid arc of >1 quadrant.[40,41] Lipid length was measured on the longitudinal reconstructed view. Lipid index was calculated as the product of mean lipid arc and lipid length.[42] Fibrous cap thickness (FCT) was measured 3 times at the thinnest point, and the average value was calculated. Thin-cap fibroatheroma (TCFA) was defined as a lipid-rich plaque with thinnest FCT <65 µm. [3,40] Cholesterol crystals were identified as thin and linear regions of high signal intensity with high backscattering within a plaque.[41,42] Calcification was defined as a signal-poor or heterogeneous region with a sharply delineated border.[41] Thrombus was defined as an irregular mass with minimum diameter of at least 250 µm adherent to the vessel wall or floating within the lumen. [3,41] Thrombus was classified into red thrombus (identified by high backscattering with high signal attenuation) or white thrombus (identified by homogeneous backscattering with low signal attenuation).[44,45] The reference lumen area was defined as the mean of the most normal appearing segments 5 mm proximal and distal to the lesion shoulders by OCT. Percent area stenosis was calculated using the formula: (reference lumen area - minimum lumen area) / reference lumen area × 100.[41]

Table S1. Determinants of Clinical Presentation, Angiographic Findings, and OCT Findings (Multivariate Logistic Regression Analysis)

**STEMI (NSTE-ACS as reference)**			
	OR	95% CI	p Value
Age, years	0.98	0.96-1.00	0.051
Male	0.97	0.51-1.84	0.929
Current smoking	1.37	0.81-2.33	0.227
Hypertension	0.56	0.34-0.93	0.021
Dyslipidemia	0.81	0.48-1.35	0.406
Diabetes mellitus	0.68	0.33-1.41	0.292
Chronic kidney disease	1.45	0.61-3.47	0.394
Previous MI	0.83	0.19-3.71	0.803
Previous PCI	0.26	0.07-1.05	0.054
eGFR, ml/min/1.73 m^2	0.99	0.97-1.00	0.031
Total cholesterol, mg/dl	0.95	0.93-0.96	<0.001
LDL-C, mg/dl	1.06	1.04-1.07	<0.001
HDL-C, mg/dl	1.05	1.02-1.08	<0.001
Triglycerides, mg/dl	1.00	1.00-1.01	0.002
HbA1c, %	1.03	0.80-1.32	0.838
**Initial TIMI flow ≤1**			
	OR	95% CI	p Value
Age, years	0.99	0.97-1.01	0.174
Male	0.61	0.32-1.15	0.122
Current smoking	1.49	0.89-2.49	0.122
Hypertension	0.82	0.50-1.33	0.403
Dyslipidemia	0.82	0.49-1.37	0.438
Diabetes mellitus	0.84	0.40-1.76	0.641
Chronic kidney disease	1.28	0.56-2.90	0.551
Previous MI	0.76	0.17-3.41	0.715
Previous PCI	0.30	0.07-1.27	0.096
eGFR, ml/min/1.73 m^2	0.99	0.98-1.00	0.196
Total cholesterol, mg/dl	0.96	0.95-0.98	<0.001
LDL-C, mg/dl	1.04	1.03-1.06	<0.001
HDL-C, mg/dl	1.05	1.02-1.07	<0.001
Triglycerides, mg/dl	1.00	1.00-1.01	0.017
HbA1c, %	1.14	0.89-1.47	0.286
**Diameter stenosis >70%**			
	OR	95% CI	p Value
Age, years	1.04	1.01-1.06	<0.001
Male	0.79	0.40-1.55	0.486
Current smoking	0.61	0.37-1.03	0.059
Hypertension	0.92	0.56-1.51	0.739

Dyslipidemia	2.03	1.22-3.36	0.005
Diabetes mellitus	1.31	0.62-2.77	0.447
Chronic kidney disease	1.95	0.81-4.70	0.129
Previous MI	1.81	0.44-7.50	0.403
Previous PCI	0.48	0.14-1.65	0.235
eGFR, ml/min/1.73 m^2	1.01	1.00-1.03	0.027
Total cholesterol, mg/dl	0.99	0.98-1.01	0.418
LDL-C, mg/dl	1.01	1.00-1.03	0.042
HDL-C, mg/dl	0.98	0.96-1.01	0.144
Triglycerides, mg/dl	1.00	1.00-1.00	0.531
HbA1c, %	0.90	0.70-1.16	0.421
**Lipid-rich plaque**			
	OR	95% CI	p Value
Age, years	1.03	1.01-1.05	0.008
Male	0.65	0.36-1.16	0.138
Current smoking	1.26	0.79-2.03	0.318
Hypertension	0.85	0.54-1.33	0.470
Dyslipidemia	0.76	0.48-1.22	0.246
Diabetes mellitus	1.46	0.75-2.86	0.259
Chronic kidney disease	1.08	0.51-2.29	0.840
Previous MI	1.10	0.31-3.83	0.881
Previous PCI	1.40	0.45-4.40	0.555
eGFR, ml/min/1.73 m^2	1.01	0.99-1.02	0.308
Total cholesterol, mg/dl	1.00	0.99-1.01	0.993
LDL-C, mg/dl	1.00	0.99-1.01	0.505
HDL-C, mg/dl	0.99	0.96-1.01	0.172
Triglycerides, mg/dl	1.00	1.00-1.00	0.536
HbA1c, %	0.93	0.73-1.17	0.518
**Cholesterol crystal**			
	OR	95% CI	p Value
Age, years	1.02	0.99-1.05	0.182
Male	1.08	0.47-2.50	0.851
Current smoking	0.77	0.39-1.53	0.449
Hypertension	1.20	0.63-2.31	0.569
Dyslipidemia	1.39	0.67-2.87	0.365
Diabetes mellitus	0.83	0.33-2.13	0.696
Chronic kidney disease	1.01	0.34-2.98	0.982
Previous MI	1.16	0.26-5.20	0.848
Previous PCI	2.70	0.68-10.67	0.148
eGFR, ml/min/1.73 m^2	1.00	0.98-1.02	0.933
Total cholesterol, mg/dl	1.00	0.99-1.02	0.585
LDL-C, mg/dl	1.01	0.99-1.02	0.585
HDL-C, mg/dl	0.96	0.93-0.99	0.015

	OR	95% CI	p Value
Triglycerides, mg/dl	0.99	0.99-1.00	0.009
HbA1c, %	1.21	0.90-1.64	0.199
**Calcification**			
	OR	95% CI	p Value
Age, years	1.04	1.01-1.06	0.001
Male	1.11	0.60-2.04	0.737
Current smoking	0.72	0.43-1.19	0.184
Hypertension	1.31	0.81-2.13	0.262
Dyslipidemia	1.06	0.64-1.77	0.806
Diabetes mellitus	1.03	0.50-2.09	0.943
Chronic kidney disease	1.21	0.54-2.69	0.641
Previous MI	0.30	0.07-1.34	0.108
Previous PCI	1.35	0.39-4.63	0.625
eGFR, ml/min/1.73 m^2	1.00	0.99-1.02	0.504
Total cholesterol, mg/dl	1.00	0.99-1.01	0.879
LDL-C, mg/dl	1.00	0.99-1.02	0.540
HDL-C, mg/dl	1.00	0.97-1.02	0.783
Triglycerides, mg/dl	1.00	0.99-1.00	0.104
HbA1c, %	1.03	0.80-1.33	0.803
**Thrombus**			
	OR	95% CI	p Value
Age, years	0.95	0.92-0.97	<0.001
Male	0.63	0.30-1.31	0.205
Current smoking	1.20	0.64-2.24	0.560
Hypertension	0.51	0.28-0.93	0.026
Dyslipidemia	0.73	0.40-1.34	0.302
Diabetes mellitus	0.79	0.33-1.88	0.583
Chronic kidney disease	1.18	0.42-3.33	0.746
Previous MI	0.40	0.07-2.15	0.273
Previous PCI	1.51	0.29-7.80	0.615
eGFR, ml/min/1.73 m^2	0.97	0.95-0.98	<0.001
Total cholesterol, mg/dl	0.96	0.95-0.98	<0.001
LDL-C, mg/dl	1.04	1.02-1.06	<0.001
HDL-C, mg/dl	1.04	1.01-1.08	0.004
Triglycerides, mg/dl	1.00	1.00-1.00	0.468
HbA1c, %	1.20	0.85-1.71	0.291

CI = confidence interval; eGFR = estimated glomerular filtration; LDL-C = low-density lipoprotein-cholesterol; HbA1c = hemoglobin A1c; HDL-C = high-density lipoprotein-cholesterol; MI = myocardial infarction; NSTE-ACS = non-ST-segment elevation acute coronary syndrome; OR = odds ratio; PCI = percutaneous coronary intervention; STEMI = ST-segment elevation myocardial infarction

Table S2. Determinants of Angiographic and OCT Findings (Multivariate Linear Regression Analysis)

**Minimum lumen diameter, mm**		
	Beta (95% CI)	p Value
Age, years	-0.01 (-0.02, -0.01)	<0.001
Male	0.02 (-0.15, 0.19)	0.817
Current smoking	0.10 (-0.03, 0.24)	0.129
Hypertension	-0.04 (-0.17, 0.09)	0.564
Dyslipidemia	-0.21 (-0.35, -0.08)	0.002
Diabetes mellitus	-0.10 (-0.29, 0.09)	0.312
Chronic kidney disease	-0.15 (-0.37, 0.06)	0.163
Previous MI	-0.28 (-0.64, 0.08)	0.130
Previous PCI	0.55 (0.22, 0.88)	0.001
eGFR, ml/min/1.73 m^2	0.00 (-0.01, 0.00)	0.267
Total cholesterol, mg/dl	0.00 (0.00, 0.01)	0.080
LDL-C, mg/dl	0.00 (-0.01, 0.00)	0.003
HDL-C, mg/dl	0.00 (0.00, 0.01)	0.301
Triglycerides, mg/dl	0.00 (0.00, 0.00)	0.628
HbA1c, %	0.05 (-0.02, 0.12)	0.130
**Reference lumen diameter, mm**		
	Beta (95% CI)	p Value
Age, years	-0.01 (-0.01, 0.00)	0.002
Male	0.08 (-0.10, 0.26)	0.373
Current smoking	0.05 (-0.10, 0.19)	0.510
Hypertension	-0.07 (-0.20, 0.07)	0.343
Dyslipidemia	-0.12 (-0.26, 0.03)	0.106
Diabetes mellitus	-0.13 (-0.34, 0.07)	0.211
Chronic kidney disease	-0.07 (-0.30, 0.16)	0.547
Previous MI	-0.38 (-0.76, 0.01)	0.054
Previous PCI	0.50 (0.15, 0.85)	0.006
eGFR, ml/min/1.73 m^2	0.00 (0.00, 0.00)	0.920
Total cholesterol, mg/dl	0.00 (-0.01, 0.00)	0.275
LDL-C, mg/dl	0.00 (0.00, 0.00)	0.567
HDL-C, mg/dl	0.00 (0.00-0.01)	0.393
Triglycerides, mg/dl	0.00 (0.00, 0.00)	0.133
HbA1c, %	0.02 (-0.05-0.09)	0.544
**Diameter stenosis, %**		
	Beta (95% CI)	p Value
Age, years	0.32 (0.16, 0.48)	<0.001
Male	-0.59 (-5.49, 4.32)	0.814
Current smoking	-2.74 (-6.68, 1.19)	0.172
Hypertension	0.73 (-3.04, 4.49)	0.705

Dyslipidemia	5.87 (1.94, 9.80)	0.004
Diabetes mellitus	1.82 (-3.81, 7.44)	0.526
Chronic kidney disease	4.39 (-1.96, 10.75)	0.175
Previous MI	4.65 (-5.96, 15.27)	0.389
Previous PCI	-12.94 (-22.67, -3.22)	0.009
eGFR, ml/min/1.73 m^2	0.06 (-0.04, 0.16)	0.210
Total cholesterol, mg/dl	-0.10 (-0.19, 0.00)	0.047
LDL-C, mg/dl	0.16 (0.07, 0.26)	<0.001
HDL-C, mg/dl	-0.11 (-0.28, 0.07)	0.238
Triglycerides, mg/dl	0.01 (-0.01, 0.03)	0.594
HbA1c, %	-0.74 (-2.70, 1.22)	0.459
**Minimum lumen area, mm^2**		
	Beta (95% CI)	p Value
Age, years	-0.02 (-0.03, -0.01)	<0.001
Male	0.18 (-0.14, 0.50)	0.268
Current smoking	-0.10 (-0.36, 0.15)	0.434
Hypertension	0.06 (-0.19, 0.30)	0.653
Dyslipidemia	-0.16 (-0.42, 0.09)	0.206
Diabetes mellitus	-0.30 (-0.66, 0.07)	0.109
Chronic kidney disease	-0.10 (-0.52, 0.31)	0.619
Previous MI	-0.29 (-0.99, 0.41)	0.420
Previous PCI	0.36 (-0.30, 1.02)	0.279
eGFR, ml/min/1.73 m^2	0.00 (-0.01, 0.00)	0.190
Total cholesterol, mg/dl	-0.01 (-0.01, 0.00)	0.015
LDL-C, mg/dl	0.00 (0.00, 0.01)	0.117
HDL-C, mg/dl	0.02 (0.01, 0.03)	0.002
Triglycerides, mg/dl	0.00 (0.00, 0.00)	0.016
HbA1c, %	0.13 (0.00, 0.26)	0.045
**Mean lipid arc, °**		
	Beta (95% CI)	p Value
Age, years	1.41 (0.53, 2.29)	0.002
Male	-4.33 (-28.81, 20.14)	0.727
Current smoking	-5.39 (-25.46, 14.67)	0.596
Hypertension	0.29 (-19.52, 20.09)	0.977
Dyslipidemia	-5.72 (-26.23, 14.79)	0.582
Diabetes mellitus	-14.72 (-42.9, 13.46)	0.306
Chronic kidney disease	-16.58 (-46.87, 13.70)	0.281
Previous MI	-1.97 (-56.78, 52.85)	0.944
Previous PCI	30.10 (-20.92, 81.12)	0.245
eGFR, ml/min/1.73 m^2	0.24 (-0.28, 0.75)	0.363
Total cholesterol, mg/dl	0.52 (-0.05, 1.10)	0.073
LDL-C, mg/dl	-0.47 (-0.99, 0.06)	0.084
HDL-C, mg/dl	-0.42 (-1.44, 0.61)	0.425

Triglycerides, mg/dl	0.01 (-0.11, 0.13)	0.846
HbA1c, %	8.54 (-1.98, 19.06)	0.111
**Lipid length, mm**		
	Beta (95% CI)	p Value
Age, years	0.12 (0.07, 0.17)	<0.001
Male	0.31 (-1.13, 1.74)	0.672
Current smoking	0.53 (-0.65, 1.71)	0.373
Hypertension	0.49 (-0.67, 1.65)	0.405
Dyslipidemia	0.81 (-0.39, 2.01)	0.186
Diabetes mellitus	1.29 (-0.36, 2.95)	0.124
Chronic kidney disease	0.34 (-1.44, 2.11)	0.707
Previous MI	1.93 (-1.28, 5.15)	0.237
Previous PCI	-0.94 (-3.93, 2.06)	0.537
eGFR, ml/min/1.73 m^2	-0.03 (-0.06, 0.00)	0.088
Total cholesterol, mg/dl	0.04 (0.01, 0.07)	0.020
LDL-C, mg/dl	-0.03 (-0.06, 0.00)	0.099
HDL-C, mg/dl	-0.05 (-0.11, 0.01)	0.091
Triglycerides, mg/dl	0.00 (-0.01, 0.01)	0.842
HbA1c, %	-0.40 (-1.02, 0.21)	0.199
**Lipid index, °mm**		
	Beta (95% CI)	p Value
Age, years	38.95 (23.14, 54.75)	<0.001
Male	-18.98 (-458.91, 420.96)	0.932
Current smoking	173.51 (-187.14, 534.17)	0.343
Hypertension	75.49 (-280.47, 431.46)	0.676
Dyslipidemia	151.36 (-217.24, 519.97)	0.418
Diabetes mellitus	162.25 (-344.22, 668.71)	0.528
Chronic kidney disease	-39.52 (-583.90, 504.86)	0.886
Previous MI	425.30 (-560.01, 1410.60)	0.395
Previous PCI	75.36 (-841.69, 992.41)	0.872
eGFR, ml/min/1.73 m^2	-5.10 (-14.39, 4.18)	0.279
Total cholesterol, mg/dl	13.17 (2.83-23.5)	0.013
LDL-C, mg/dl	-9.24 (-18.75, 0.27)	0.057
HDL-C, mg/dl	-15.56 (-34.03-2.90)	0.098
Triglycerides, mg/dl	-0.13 (-2.30-2.04)	0.905
HbA1c, %	-2.70 (-191.79, 186.38)	0.977

CI = confidence interval; eGFR = estimated glomerular filtration; LDL-C = low-density lipoprotein-cholesterol; HbA1c = hemoglobin A1c; HDL-C = high-density lipoprotein-cholesterol; MI = myocardial infarction; PCI = percutaneous coronary intervention

Table S3. Clinical, Angiographic, and OCT Findings in Patients With STEMI

	Age (years)					
	<45 (n = 34)	45–54 (n = 66)	55–64 (n = 91)	65–74 (n = 77)	≥75 (n = 30)	*p* value
Male	32 (94.1)	56 (84.8)	77 (84.6)	60 (77.9)	20 (66.7)	0.003
BMI, kg/m^2	25.8 ± 4.0	25.1 ± 3.7	25.0 ± 2.8	24.0 ± 3.9	23.3 ± 2.9	<0.001
Current smoking	23 (67.6)	44 (66.7)	58 (63.7)	33 (42.9)	3 (10.0)	<0.001
Hypertension	13 (38.2)	19 (28.8)	40 (44.0)	43 (55.8)	18 (60.0)	0.001
Dyslipidemia	9 (26.5)	32 (48.5)	42 (46.2)	44 (57.1)	18 (60.0)	0.004
Diabetes mellitus	5 (14.7)	13 (19.7)	21 (23.1)	14 (18.2)	8 (26.7)	0.428
Chronic kidney disease	3 (8.8)	8 (12.1)	7 (7.7)	12 (15.6)	6 (20.0)	0.139
Previous MI	0 (0.0)	1 (1.5)	4 (4.4)	3 (3.9)	1 (3.3)	0.253
Previous PCI	0 (0.0)	0 (0.0)	6 (6.6)	2 (2.6)	2 (6.7)	0.101
Previous CABG	0 (0.0)	0 (0.0)	0 (0.0)	0 (0.0)	0 (0.0)	>0.999
**Medication**						
Aspirin	2 (20.0)	1 (2.4)	11 (21.2)	5 (9.4)	2 (8.0)	0.856
P2Y12 inhibitor	0 (0.0)	1 (2.4)	4 (7.7)	2 (3.8)	2 (8.0)	0.371
Statin	0 (0.0)	1 (2.4)	7 (13.5)	9 (17.0)	3 (12.0)	0.046
Beta blocker	1 (10.0)	2 (4.9)	6 (11.5)	7 (13.2)	1 (4.0)	0.809
ACE-I or ARB	1 (10.0)	5 (12.2)	18 (34.6)	10 (18.9)	7 (28.0)	0.232
CCI	2 (40.0)	3 (8.3)	15 (31.9)	13 (26.0)	11 (50.0)	0.013
**Laboratory data**						
eGFR, ml/min/1.73 m^2	74.6 ± 25.2	69.7 ± 20.0	69.6 ± 16.5	69.0 ± 19.5	64.8 ± 18.2	0.270
Total cholesterol, mg/dl	171.8 ± 41.9	186.8 ± 38.1	179.9 ± 43.6	192.5 ± 38.9	178.1 ± 31.9	0.269
LDL-C, mg/dl	110.9 ± 41.2	121.9 ± 35.8	120.6 ± 41.7	131.0 ± 37.4	121.9 ± 37.5	0.100
HDL-C, mg/dl	45.9 ± 11.4	47.8 ± 12.0	45.0 ± 11.8	48.5 ± 12.4	48.2 ± 11.0	0.268
Triglycerides, mg/dl	117.0 (60.7–176.3)	111.0 (67.8–159.7)	115.2 (81.1–152.0)	113.5 (64.5–165.0)	69.1 (56.0–103.0)	0.049
HbA1c, %	6.0 ± 1.0	6.2 ± 1.4	6.3 ± 1.4	6.0 ± 0.7	6.1 ± 1.1	0.972
Hs-CRP, mg/dl	0.64 (0.15–1.19)	0.20 (0.05–0.56)	0.29 (0.09–0.80)	0.12 (0.03–0.42)	0.20 (0.05–0.50)	0.007
Hemoglobin, g/dl	15.5 ± 1.8	14.5 ± 1.5	14.7 ± 1.7	14.2 ± 1.8	13.5 ± 2.0	0.008

Peak CK-MB, IU/l	163.4 (62.0–274.0)	157.1 (78.0–284.3)	179.4 (94.8–322.8)	249.0 (139.3–436.5)	150.9 (114.3–278.0)	0.149
LVEF, %	57.2 ± 7.5	54.9 ± 10.8	54.6 ± 10.2	56.0 ± 11.2	55.8 ± 11.2	0.994
**Angiographic findings**						
Infarct-related artery						0.435*
RCA	9 (26.5)	18 (27.3)	28 (30.8)	32 (41.6)	9 (30.0)	
LAD	24 (70.6)	40 (60.6)	54 (59.3)	36 (46.8)	18 (60.0)	
LCx	1 (2.9)	8 (12.1)	9 (9.9)	9 (11.7)	3 (10.0)	
Culprit lesion site						0.798*
Proximal segment	15 (44.1)	26 (39.4)	41 (46.1)	34 (45.3)	13 (43.3)	
Mid segment	10 (29.4)	29 (43.9)	28 (31.5)	27 (36.0)	9 (30.0)	
Distal segment	9 (26.5)	11 (16.7)	20 (22.5)	14 (18.7)	8 (26.7)	
Multivessel disease	12 (35.3)	22 (33.3)	31 (34.8)	31 (41.3)	10 (33.3)	0.628
Initial TIMI flow ≤1	18 (52.9)	37 (56.1)	52 (58.4)	42 (56.0)	14 (46.7)	0.720
MLD, mm	1.48 ± 0.80	0.70 ± 0.68	0.71 ± 0.61	0.48 ± 0.57	0.38 ± 0.55	<0.001
RVD, mm	3.64 ± 0.66	2.94 ± 0.61	3.06 ± 0.66	2.85 ± 0.51	2.79 ± 0.75	<0.001
Lesion length, mm	14.7 ± 5.7	15.1 ± 6.7	14.9 ± 6.5	14.9 ± 6.7	16.0 ± 5.1	0.506
Diameter stenosis, %	59.3 ± 19.4	77.3 ± 21.0	77.1 ± 19.0	84.0 ± 17.7	88.3 ± 13.9	<0.001
Diameter stenosis >70%	5 (15.2)	37 (56.9)	53 (60.2)	55 (73.3)	26 (86.7)	<0.001
**OCT findings**						
Lipid-rich plaque	7 (20.6)	23 (34.8)	43 (47.3)	41 (53.2)	18 (60.0)	<0.001
TCFA	0 (0.0)	4 (6.1)	8 (8.8)	6 (7.8)	2 (6.7)	0.326
Cholesterol crystal	1 (2.9)	7 (10.6)	14 (15.4)	12 (15.6)	9 (30.0)	0.007
Calcification	0 (0.0)	21 (31.8)	19 (20.9)	29 (37.7)	17 (56.7)	<0.001
Thrombus	33 (97.1)	63 (95.5)	81 (89.0)	71 (92.2)	26 (86.7)	0.107
White	29 (87.9)	46 (73.0)	69 (85.2)	57 (80.3)	20 (76.9)	0.776
Red	4 (12.1)	17 (27.0)	12 (14.8)	14 (19.7)	6 (23.1)	
Minimum lumen area, mm^2	2.91 (1.52–3.71)	1.27 (0.92–1.79)	1.20 (0.92–1.73)	1.00 (0.80–1.50)	0.90 (0.75–1.18)	<0.001
Reference lumen area, mm^2	8.73 (6.73–10.36)	6.40 (4.69–8.02)	6.75 (5.21–8.38)	6.11 (4.83–7.59)	5.70 (4.50–7.57)	<0.001

Area stenosis, %	71.1 (61.3–80.0)	80.3 (70.7–84.8)	80.8 (71.3–85.0)	81.4 (75.3–85.2)	82.8 (68.8–87.5)	0.001
Minimum fibrous cap thickness, μm	130.0 (91.7–135.0)	86.7 (66.0–122.5)	100.0 (77.0–120.0)	100.0 (80.0–120.8)	90.0 (80.0–115.3)	0.928
Mean lipid arc, °	173.1 (156.7–193.0)	191.8 (155.8–250.6)	204.0 (182.8–251.6)	199.4 (154.1–256.3)	264.4 (235.0–295.8)	0.010
Lipid length, mm	8.1 (7.0–8.8)	5.2 (3.4–7.5)	7.4 (5.2–9.5)	8.7 (6.4–10.1)	10.0 (8.1–14.1)	<0.001
Lipid index, °mm	1248.9 (1172.0–1534.7)	1007.8 (566.3– 1720.9)	1466.6 (1083.3–2134.4)	1552.4 (1202.0–2431.1)	2564.7 (2208.6–3595.5)	<0.001

Values shown are n (%), mean (standard deviation, or median (25th–75th percentile). *p* values are for the Jonckheere-Terpstra trend test for continuous variables or the Cochran-Armitage trend test for categorical data. **p* value for Chi-square test. Medication data were analyzed only in available cases. Angiographic data except infarct-related artery were missing in 4 (1.4%) cases.

Abbreviations: ACE-I = angiotensin-converting enzyme inhibitor; ARB = angiotensin II receptor blocker; BMI = body mass index; CABG = coronary artery bypass graft; CCI = calcium channel inhibitor; CK = creatine kinase; eGFR = estimated glomerular filtration rate; HbA1c = hemoglobin A1c; HDL = high-density lipoprotein; Hs-CRP = high-sensitivity C-reactive protein; LAD = left anterior descending artery; LCx = left circumflex artery; LDL = low-density lipoprotein; LVEF = left ventricular ejection fraction; MI = myocardial infarction; MLD = minimum lumen diameter; OCT = optical coherence tomography; PCI = percutaneous coronary intervention; RCA = right coronary artery; RVD = reference vessel diameter; TCFA = thin cap fibroatheroma; TIMI = Thrombolysis in Myocardial Infarction.

SI conversion factor: To convert cholesterol levels to millimoles per liter, multiply by 0.0259; C-reactive protein to nanomoles per liter, multiply by 9.524; Hemoglobin to millimoles per liter, multiply by 0.6206; triglycerides to millimoles per liter, multiply by 0.0113.

Table S4. Clinical, Angiographic, and OCT Findings in Patients With AMI

	Age (years)					
	<45 (n = 46)	45–54 (n = 96)	55–64 (n = 132)	65–74 (n = 131)	≥75 (n = 73)	*p* value
Male	43 (93.5)	84 (87.5)	108 (81.8)	100 (76.3)	50 (68.5)	<0.001
BMI, kg/m^2	25.9 ± 3.9	25.3 ± 3.7	25.2 ± 2.7	24.1 ± 3.8	23.8 ± 3.1	<0.001
Current smoking	33 (71.7)	61 (63.5)	78 (59.1)	52 (39.7)	12 (16.4)	<0.001
Hypertension	17 (37.0)	35 (36.5)	65 (49.2)	78 (59.5)	52 (71.2)	<0.001
Dyslipidemia	16 (34.8)	57 (59.4)	70 (53.0)	75 (57.3)	46 (63.0)	0.027
Diabetes mellitus	7 (15.2)	20 (20.8)	31 (23.5)	30 (22.9)	25 (34.2)	0.025
Chronic kidney disease	3 (6.5)	10 (10.4)	10 (7.6)	20 (15.3)	15 (20.5)	0.007
Previous MI	1 (2.2)	3 (3.1)	6 (4.5)	4 (3.1)	5 (6.8)	0.286
Previous PCI	0 (0.0)	2 (2.1)	11 (8.3)	4 (3.1)	5 (6.8)	0.150
Previous CABG	0 (0.0)	0 (0.0)	1 (0.8)	1 (0.8)	0 (0.0)	0.711
Clinical presentation						<0.001
STEMI	34 (73.9)	66 (68.8)	91 (68.9)	77 (58.8)	40 (41.1)	
NSTEMI	12 (26.1)	30 (31.2)	41 (31.1)	54 (41.2)	43 (58.9)	
**Medication**						
Aspirin	3 (18.8)	6 (9.2)	18 (22.8)	13 (14.9)	7 (13.2)	0.996
P2Y12 inhibitor	1 (6.2)	4 (6.2)	8 (10.1)	7 (8.0)	3 (5.7)	0.946
Statin	1 (6.2)	9 (13.8)	17 (21.5)	18 (20.7)	8 (15.4)	0.403
Beta blocker	3 (18.8)	6 (9.2)	13 (16.7)	15 (17.2)	4 (7.5)	0.752
ACE-I or ARB	2 (12.5)	15 (23.1)	28 (35.4)	22 (25.3)	21 (39.6)	0.063
CCI	2 (25.0)	7 (12.5)	20 (28.6)	21 (25.9)	21 (42.0)	0.004
**Laboratory data**						
eGFR, ml/min/1.73 m^2	76.6 ± 24.1	72.7 ± 27.9	70.2 ± 20.2	69.2 ± 20.5	67.1 ± 24.9	0.024
Total cholesterol, mg/dl	181.2 ± 45.4	194.3 ± 47.0	183.5 ± 43.9	193.6 ± 40.8	184.0 ± 39.8	0.747
LDL-C, mg/dl	117.7 ± 41.6	127.0 ± 42.2	120.9 ± 42.4	127.3 ± 37.9	116.5 ± 37.3	0.835
HDL-C, mg/dl	46.3 ± 13.6	48.0 ± 12.6	46.3 ± 15.0	48.7 ± 14.0	48.9 ± 11.1	0.085
Triglycerides, mg/dl	118.7 (52.7–189.7)	116.0 (67.8–167.3)	119.0 (79.7–159.5)	104.0 (64.7–165.0)	87.0 (60.0–133.5)	0.019

HbA1c, %	6.3 ± 1.9	6.2 ± 1.4	6.2 ± 1.3	6.0 ± 0.8	6.2 ± 1.1	0.359
Hs-CRP, mg/dl	0.36 (0.10–1.07)	0.20 (0.07–0.54)	0.24 (0.08–0.70)	0.13 (0.03–0.49)	0.30 (0.06–0.75)	0.236
Hemoglobin, g/dl	15.2 ± 1.6	14.6 ± 1.5	14.4 ± 1.6	14.2 ± 1.7	13.4 ± 2.0	<0.001
Peak CK-MB, IU/l	104.7 (22.0–256.9)	127.0 (44.0–257.5)	129.0 (28.3–259.5)	128.5 (24.0–300.0)	84.0 (25.5–188.0)	0.478
LVEF, %	57.9 ± 7.3	56.3 ± 10.7	55.5 ± 10.1	57.0 ± 11.1	55.7 ± 12.2	0.812
**Angiographic findings**						
Infarct-related artery						0.273*
RCA	10 (21.7)	26 (27.1)	35 (26.5)	49 (37.4)	20 (27.4)	
LAD	31 (67.4)	55 (57.3)	80 (60.6)	60 (45.8)	41 (56.2)	
LCx	5 (10.9)	15 (15.6)	17 (12.9)	22 (16.8)	12 (16.4)	
Culprit lesion site						0.913*
Proximal segment	18 (39.1)	36 (39.1)	61 (46.9)	57 (44.5)	31 (43.1)	
Mid segment	16 (34.8)	37 (40.2)	39 (30.0)	46 (35.9)	22 (30.6)	
Distal segment	12 (26.1)	19 (20.7)	30 (23.1)	25 (19.5)	19 (26.4)	
Multivessel disease	13 (28.3)	25 (27.2)	44 (34.1)	48 (37.8)	26 (36.1)	0.105
Initial TIMI flow ≤1	19 (41.3)	43 (46.7)	58 (44.6)	50 (39.1)	17 (23.6)	0.012
MLD, mm	1.41 ± 0.80	0.68 ± 0.62	0.75 ± 0.65	0.59 ± 0.55	0.56 ± 0.53	<0.001
RVD, mm	3.59 ± 0.72	2.83 ± 0.60	3.03 ± 0.67	2.83 ± 0.54	2.68 ± 0.68	<0.001
Lesion length, mm	14.7 ± 5.6	14.9 ± 7.3	15.0 ± 6.5	14.6 ± 6.2	15.8 ± 6.3	0.296
Diameter stenosis, %	60.7 ± 19.3	76.7 ± 19.9	75.8 ± 19.4	80.0 ± 17.2	79.2 ± 19.3	<0.001
Diameter stenosis >70%	12 (26.7)	55 (60.4)	75 (59.5)	90 (72.0)	48 (66.7)	<0.001
**OCT findings**						
Lipid-rich plaque	12 (26.1)	32 (33.3)	61 (46.2)	60 (45.8)	40 (54.8)	<0.001
TCFA	1 (2.2)	6 (6.3)	11 (8.3)	7 (5.3)	9 (12.3)	0.157
Cholesterol crystal	1 (2.2)	10 (10.4)	21 (15.9)	22 (16.8)	18 (24.7)	0.001
Calcification	2 (4.3)	29 (30.2)	30 (22.7)	47 (35.9)	42 (57.5)	<0.001
Thrombus	43 (93.5)	88 (91.7)	110 (83.3)	99 (75.6)	50 (68.5)	<0.001
White	33 (76.7)	68 (77.3)	93 (84.5)	84 (84.8)	36 (72.0)	0.841
Red	10 (23.3)	20 (22.7)	17 (15.5)	15 (15.2)	14 (28.0)	

Minimum lumen area, mm^2	2.91 (1.44–3.81)	1.20 (0.84–1.88)	1.15 (0.80–1.70)	0.91 (0.80–1.50)	0.90 (0.70–1.26)	<0.001
Reference lumen area, mm^2	9.14 (6.73–11.04)	6.38 (4.70–8.04)	6.58 (5.15–8.13)	5.90 (4.65–7.76)	5.75 (3.77–7.36)	<0.001
Area stenosis, %	74.3 (60.7–81.6)	80.5 (70.9–85.6)	81.2 (72.5–87.1)	81.2 (74.7–87.1)	82.0 (71.3–88.5)	0.001
Minimum fibrous cap thickness, µm	130.0 (83.2–156.5)	90.0 (70.0–133.2)	97.0 (77.0–130.0)	102.0 (80.0–128.3)	101.5 (80.0–126.0)	0.898
Mean lipid arc, °	196.1 (173.1–245.3)	192.8 (160.9–249.0)	213.2 (184.2–262.2)	202.6 (163.5–266.9)	259.7 (212.6–276.7)	0.010
Lipid length, mm	7.4 (6.7–9.1)	6.6 (3.7–8.3)	7.6 (5.1–9.7)	8.7 (6.0–10.3)	10.0 (7.7–12.5)	<0.001
Lipid index, °mm	1294.3 (1176.6–1889.5)	1185.4 (714.0–1730.9)	1538.8 (1105.6–2441.1)	1553.8 (1204.8–2438.8)	2423.0 (1849.5–3129.1)	<0.001

Values shown are n (%), mean (standard deviation, or median (25th–75th percentile). *p* values are for the Jonckheere-Terpstra trend test for continuous variables or the Cochran-Armitage trend test for categorical data. $^{*}p$ value for Chi-square test. Medication data were analyzed only in available cases. Angiographic data except infarct-related artery were missing in 10 (2.1%) cases.

Abbreviations: ACE-I = angiotensin-converting enzyme inhibitor; AMI = acute myocardial infarction; ARB = angiotensin II receptor blocker; BMI = body mass index; CABG = coronary artery bypass graft; CCI = calcium channel inhibitor; CK = creatine kinase; eGFR = estimated glomerular filtration rate; HbA1c = hemoglobin A1c; HDL = high-density lipoprotein; Hs-CRP = high-sensitivity C-reactive protein; LAD = left anterior descending artery; LCx = left circumflex artery; LDL = low-density lipoprotein; LVEF = left ventricular ejection fraction; MI = myocardial infarction; MLD = minimum lumen diameter; NSTEMI = non-ST-segment elevation myocardial infarction; OCT = optical coherence tomography; PCI = percutaneous coronary intervention; RCA = right coronary artery; RVD = reference vessel diameter; STEMI = ST-segment elevation myocardial infarction; TCFA = thin cap fibroatheroma; TIMI = Thrombolysis in Myocardial Infarction.

SI conversion factor: To convert cholesterol levels to millimoles per liter, multiply by 0.0259; C-reactive protein to nanomoles per liter, multiply by 9.524; Hemoglobin to millimoles per liter, multiply by 0.6206; triglycerides to millimoles per liter, multiply by 0.0113.

Table S5. Clinical, Angiographic, and OCT Findings in Patients with Coronary Thrombus

	Age (years)					
	<45 (n = 49)	45–54 (n = 105)	55–64 (n = 119)	65–74 (n = 114)	≥75 (n = 54)	p Value
Male	45 (91.8)	91 (86.7)	99 (83.2)	80 (70.2)	35 (64.8)	<0.001
BMI, kg/m^2	25.8 ± 3.8	25.3 ± 3.7	25.2 ± 2.8	24.6 ± 3.9	23.2 ± 3.1	<0.001
Hypertension	18 (36.7)	42 (40.0)	52 (43.7)	66 (57.9)	36 (66.7)	<0.001
Dyslipidemia	17 (34.7)	65 (61.9)	59 (49.6)	67 (58.8)	34 (63.0)	0.041
Diabetes mellitus	7 (14.3)	24 (22.9)	29 (24.4)	29 (25.4)	19 (35.2)	0.024
Current smoking	31 (63.3)	65 (61.9)	68 (57.1)	48 (42.1)	8 (14.8)	<0.001
Chronic kidney disease	4 (8.2)	10 (9.5)	12 (10.1)	16 (14.0)	12 (22.2)	0.017
Previous MI	2 (4.1)	5 (4.8)	8 (6.7)	3 (2.6)	3 (5.6)	0.866
Previous PCI	1 (2.0)	5 (4.8)	12 (10.1)	8 (7.0)	4 (7.4)	0.223
Previous CABG	0 (0.0)	0 (0.0)	1 (0.8)	0 (0.0)	0 (0.0)	0.971
Clinical presentation						0.134
STEMI	33 (67.3)	63 (60.0)	81 (68.1)	71 (62.3)	26 (48.1)	
NSTE-ACS	16 (32.7)	42 (40.0)	38 (31.9)	43 (37.7)	28 (51.9)	
**Medication**						
Aspirin	5 (22.7)	11 (14.7)	15 (21.7)	14 (17.7)	6 (14.6)	0.745
P2Y12 inhibitor	2 (9.1)	8 (10.7)	7 (10.1)	8 (10.1)	3 (7.3)	0.722
Statin	2 (9.1)	13 (17.3)	14 (20.3)	18 (22.8)	7 (17.1)	0.366
Beta blocker	5 (22.7)	9 (12.0)	9 (13.2)	15 (19.0)	2 (4.9)	0.396
ACE-I or ARB	2 (9.1)	16 (21.3)	20 (29.0)	19 (24.1)	14 (34.1)	0.055
CCI	2 (18.2)	6 (10.9)	16 (28.6)	16 (23.9)	15 (41.7)	0.005
**Laboratory data**						
eGFR, ml/min/1.73 m^2	74.1 ± 23.8	71.9 ± 26.3	67.5 ± 20.0	66.9 ± 18.9	66.6 ± 24.8	0.014
Total cholesterol, mg/dl	176.5 ± 43.8	192.2 ± 48.8	178.1 ± 41.4	191.9 ± 40.9	187.3 ± 40.0	0.197
LDL-C, mg/dl	115.1 ± 41.7	124.1 ± 44.2	116.7 ± 42.1	126.3 ± 39.0	120.9 ± 37.2	0.376
HDL-C, mg/dl	45.2 ± 13.7	47.5 ± 12.6	46.1 ± 15.0	48.6 ± 14.0	49.5 ± 11.7	0.016

Triglycerides, mg/dl	117.0 (44.5–193.8)	105.0 (61.8–160.0)	108.1 (62.0–152.5)	102.0 (62.5–163.0)	86.5 (59.3–128.3)	0.154
HbA1c, %	6.3 ± 1.9	6.2 ± 1.3	6.3 ± 1.5	6.1 ± 1.0	6.2 ± 1.0	0.276
Hs-CRP, mg/dl	0.39 (0.11–1.07)	0.20 (0.09–0.50)	0.29 (0.08–0.86)	0.10 (0.03–0.44)	0.20 (0.05–0.52)	0.037
Hemoglobin, g/dl	15.1 ± 1.4	14.6 ± 1.6	14.5 ± 1.6	14.2 ± 1.6	13.8 ± 1.9	0.006
Peak CK-MB, IU/l	104.7 (18.8–253.0)	114.0 (34.0–257.0)	151.0 (28.7–270.2)	149.0 (24.0–387.0)	96.0 (23.5–187.0)	0.757
LVEF, %	58.4 ± 7.1	57.4 ± 11.3	55.7 ± 10.2	57.4 ± 11.0	56.8 ± 11.6	0.576
**Angiographic findings**						
Infarct-related artery						0.079
RCA	11 (22.4)	28 (26.7)	34 (28.6)	45 (39.5)	15 (27.8)	
LAD	34 (69.4)	60 (57.1)	75 (63.0)	52 (45.6)	30 (55.6)	
LCx	4 (8.2)	17 (16.2)	10 (8.4)	17 (14.9)	9 (16.7)	
Culprit lesion site						0.958
Proximal segment	21 (42.9)	38 (37.6)	54 (45.4)	49 (43.4)	25 (46.3)	
Mid segment	17 (34.7)	41 (40.6)	41 (34.5)	40 (35.4)	16 (29.6)	
Distal segment	11 (22.4)	22 (21.8)	24 (20.2)	24 (21.2)	13 (24.1)	
Multivessel disease	15 (30.6)	30 (29.7)	40 (33.9)	38 (33.6)	19 (35.8)	0.419
Initial TIMI flow ≤1	20 (40.8)	46 (45.5)	56 (47.1)	47 (41.6)	16 (29.6)	0.186
QCA data						
MLD, mm	1.42 ± 0.82	0.71 ± 0.64	0.76 ± 0.65	0.62 ± 0.67	0.54 ± 0.56	<0.001
RVD, mm	3.53 ± 0.74	2.88 ± 0.63	3.04 ± 0.63	2.81 ± 0.53	2.69 ± 0.71	<0.001
Lesion length, mm	14.5 ± 5.6	15.1 ± 7.0	15.1 ± 6.5	14.6 ± 6.7	16.1 ± 6.7	0.593
Diameter stenosis, %	59.8 ± 20.2	76.3 ± 20.7	75.8 ± 19.2	78.9 ± 20.5	80.3 ± 19.5	<0.001
Diameter stenosis >70%	13 (26.5)	63 (62.4)	66 (57.4)	75 (67.6)	38 (70.4)	<0.001
**OCT findings**						
Quantitative						
Lipid rich plaque	14 (28.6)	38 (36.2)	58 (48.7)	56 (49.1)	34 (63.0)	<0.001
TCFA	0 (0.0)	7 (6.7)	10 (8.4)	9 (7.9)	8 (14.8)	0.020
Cholesterol crystal	2 (4.1)	16 (15.2)	23 (19.3)	17 (14.9)	11 (20.4)	0.124
Calcification	2 (4.1)	32 (30.5)	31 (26.1)	39 (34.2)	27 (50.0)	<0.001

Thrombus	49 (100.0)	105 (100.0)	119 (100.0)	114 (100.0)	54 (100.0)	–
White	38 (77.6)	76 (72.4)	100 (84.0)	94 (82.5)	38 (70.4)	0.764
Red	11 (22.4)	29 (27.6)	19 (16.0)	20 (17.5)	16 (29.6)	
Quantitative						
Minimum lumen area, mm^2	2.86 (1.24–3.83)	1.18 (0.82–1.73)	1.13 (0.80–1.57)	0.90 (0.78–1.50)	0.90 (0.80–1.20)	<0.001
Reference lumen area, mm^2	8.80 (6.65–11.00)	6.40 (4.64–8.09)	6.42 (5.10–7.97)	5.84 (4.58–7.57)	5.72 (3.84–7.66)	<0.001
Area stenosis, %	74.1 (60.5–80.6)	81.6 (72.0–86.4)	81.5 (74.7–86.7)	81.6 (74.5–87.2)	82.1 (74.2–87.6)	0.002
Minimum fibrous cap thickness, µm	130.0 (82.3–153.3)	97.0 (70.0–133.2)	100.0 (77.0–133.0)	100.0 (80.0–130.0)	100.0 (72.3–131.0)	0.637
Mean lipid arc, °	183.5 (156.3–237.5)	191.8 (145.5–247.4)	212.9 (182.9–264.4)	197.1 (150.5–263.2)	259.7 (204.3–274.6)	0.006
Lipid length, mm	7.2 (4.7–8.8)	6.6 (3.7–9.3)	7.7 (5.4–9.5)	8.1 (6.0–9.5)	10.6 (8.1–14.1)	<0.001
Lipid index, °mm	1203.6 (1051.6–1614.8)	1128.0 (727.4–1806.9)	1504.5 (1094.5–2478.5)	1552.4 (1057.9–2297.7)	2451.0 (1922.1–3595.5)	<0.001

Values shown are n (%), mean (standard deviation, or median (25th–75th percentile). *p* values are for the Jonckheere-Terpstra trend test for continuous variables or the Cochran-Armitage trend test for categorical data. $^{*}p$ value for Chi-square test. Medication data were analyzed only in available cases. Angiographic data except infarct-related artery were missing in 5 (1.1%) cases.

Abbreviations: ACE-I = angiotensin-converting enzyme inhibitor; AMI = acute myocardial infarction; ARB = angiotensin II receptor blocker; BMI = body mass index; CABG = coronary artery bypass graft; CCI = calcium channel inhibitor; CK = creatine kinase; eGFR = estimated glomerular filtration rate; HbA1c = hemoglobin A1c; HDL = high-density lipoprotein; Hs-CRP = high-sensitivity C-reactive protein; LAD = left anterior descending artery; LCx = left circumflex artery; LDL = low-density lipoprotein; LVEF = left ventricular ejection fraction; MI = myocardial infarction; MLD = minimum lumen diameter; NSTE-ACS = non-ST-segment elevation-acute coronary syndrome; OCT = optical coherence tomography; PCI = percutaneous coronary intervention; RCA = right coronary artery; RVD = reference vessel diameter; STEMI = ST-segment elevation myocardial infarction; TCFA = thin cap fibroatheroma; TIMI = Thrombolysis in Myocardial Infarction.

SI conversion factor: To convert cholesterol levels to millimoles per liter, multiply by 0.0259; C-reactive protein to nanomoles per liter, multiply by 9.524; Hemoglobin to millimoles per liter, multiply by 0.6206; triglycerides to millimoles per liter, multiply by 0.0113.

Table S6. Prevalence of Thrombus After Excluding Patients Who Underwent Thrombectomy

	Age (years)					
	**<45 (n = 47)**	**45–54 (n = 90)**	**55–64 (n = 119)**	**65–74 (n = 109)**	**≥75 (n = 63)**	**p value**
Thrombus	42 (89.4)	76 (84.4)	82 (69.5)	67 (62.0)	32 (50.8)	<0.001
White	35 (83.3)	55 (72.4)	69 (84.1)	56 (83.6)	22 (68.8)	0.151
Red	7 (16.7)	21 (27.6)	13 (15.9)	11 (16.4)	10 (31.2)	

p values are for the Cochran-Armitage trend test. Values shown are n (%).

Figure S1. Study Flow Diagram

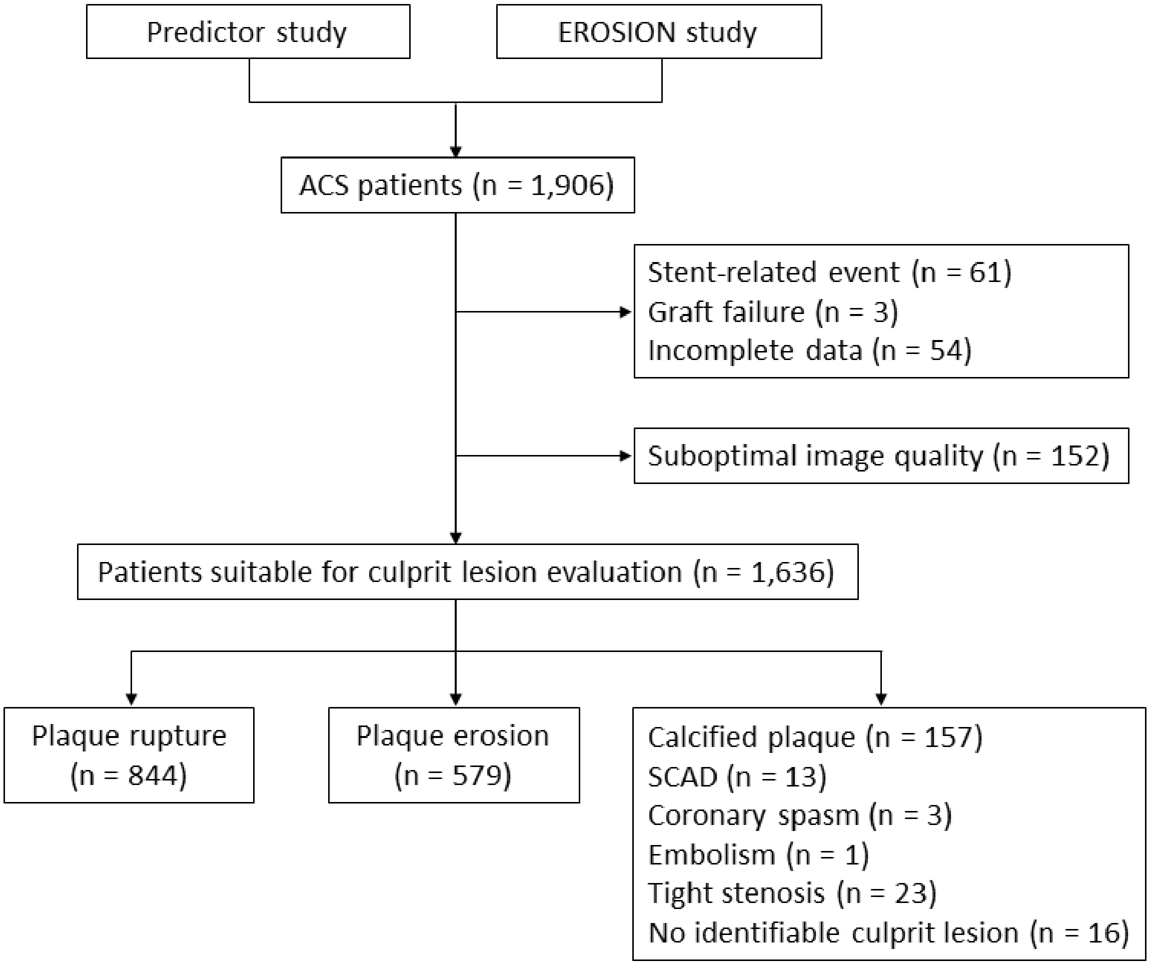

ACS = acute coronary syndromes; EROSION = Effective Anti-Thrombotic Therapy Without Stenting: Intravascular Optical Coherence Tomography–Based Management in Plaque Erosion; OCT = optical coherence tomography; PCI = percutaneous coronary intervention; SCAD = spontaneous coronary artery dissection.
